# The trehalose glycolipid C18Brar promotes antibody and T-cell immune responses to *Mannheimia haemolytica* and *Mycoplasma ovipneumoniae* whole cell antigens in sheep

**DOI:** 10.1371/journal.pone.0278853

**Published:** 2023-01-19

**Authors:** Sandeep K. Gupta, Natalie Parlane, Benjamin Bridgeman, Amy T. Lynch, Emma M. Dangerfield, Mattie S. M. Timmer, Bridget L. Stocker, D. Neil Wedlock

**Affiliations:** 1 AgResearch, Hopkirk Research Institute, Palmerston North, New Zealand; 2 School of Chemical and Physical Sciences, Victoria University of Wellington, Wellington, New Zealand; Friedrich-Alexander-Universitat Erlangen, GERMANY

## Abstract

Bronchopneumonia is a common respiratory disease in livestock. *Mannheimia haemolytica* is considered the main causative pathogen leading to lung damage in sheep, with *Mycoplasma ovipneumoniae* and ParaInfluenza virus type 3, combined with adverse physical and physiological stress, being predisposing factors. A balance of humoral and cellular immunity is thought to be important for protection against developing respiratory disease. In the current study, we compared the ability of the trehalose glycolipid adjuvant C18Brar (C18-alkylated brartemicin analogue) and three commercially available adjuvant systems *i*.*e*., Quil-A, Emulsigen-D, and a combination of Quil-A and aluminium hydroxide gel, to stimulate antibody and cellular immune responses to antigens from inactivated whole cells of *M*. *haemolytica* and *M*. *ovipneumoniae* in sheep. C18Brar and Emulsigen-D induced the strongest antigen-specific antibody responses to both *M*. *haemolytica* and *M*. *ovipneumoniae*, while C18Brar and Quil-A promoted the strongest antigen-specific IL-17A responses. The expression of genes with known immune functions was determined in antigen-stimulated blood cultures using Nanostring nCounter technology. The expression levels of *CD40*, *IL22*, *TGFB1*, and *IL2RA* were upregulated in antigen-stimulated blood cultures from animals vaccinated with C18Brar, which is consistent with T-cell activation. Collectively, the results demonstrate that C18Brar can promote both antibody and cellular responses, notably Th17 immune responses in a ruminant species.

## Introduction

Diseases of the respiratory tract, such as pneumonia, are associated with major animal welfare issues and cause considerable economic loss to livestock industries in New Zealand and world-wide [1]. These losses are often due to condemnation, downgrading of carcasses, lower growth rates, and treatment or prevention costs [2]. Bronchopneumonia is a multifactorial disease, involving interactions between different bacterial and viral pathogens, as well as predisposing factors such as host defence, environment, and stress [3]. *Mannheimia haemolytica* is the main pathogen responsible for causing lung damage, while *Mycoplasma ovipneumoniae* and ParaInfluenza virus type 3 (PI3) are considered the predisposing agents in sheep [4].

Antibody responses against *M*. *haemolytica* and *M*. *ovipneumoniae* antigens are considered essential for the development of effective vaccines against ovine pneumonia [5–8]. In the past, efforts have been made to prevent pneumonia in sheep by vaccination against leukotoxin (LKT), a secretory protein of *M*. *haemolytica* and a key virulence factor that causes cytotoxicity and apoptosis of host’s cells [9]. However, the presence of different serotypes of leukotoxin producing *M*. *haemolytica* [10, 11] makes it challenging to develop a vaccine against multi-variant leukotoxins, and vaccines based solely on *M*. *haemolytica* serotypes have shown poor efficacy [12, 13].

Cells of the respiratory tract are thought to play a crucial role in the host defence against invading respiratory pathogens. Mycoplasma species are one of the primary causative agents of chronic pneumonia in sheep [14, 15] and predominantly colonise the respiratory tract by attaching to the cilia of airway epithelial cells (AECs). Both mycoplasmas and *M*. *haemolytica* can invade broncho-epithelial cells and persist as extracellular as well as intracellular pathogens [16–20], thereby stimulating Th1 immune responses in the lungs [21–23]. In addition, Th17 cell-mediated immune responses are thought to play a key role in the pathogenesis of respiratory disease. Th17 cytokines bridge the innate and adaptive immune response in host defence against a variety of pathogens at mucosal sites by recruiting neutrophils, monocytes and other inflammatory cells that contribute to bacterial, fungal and viral clearance [24, 25]. IL-17 has been shown to play a key role in the defence against mycoplasmas and *M*. *haemolytica*, however, excessive IL-17 can result in inflammation and damage at the mucosal surface [25–29]. Accordingly, it is thought that an effective vaccine against ovine pneumonia needs to elicit a balanced humoral- and cell-mediated immune response (Th1 and Th17) against pathogens such as *M*. *ovipneumoniae* and/or *M*. *haemolytica* [30–32]. There is also evidence to suggest that a vaccine comprised of antigens from both *M*. *haemolytica* and mycoplasma may be more efficacious than a vaccine containing *M*. *haemolytica* alone in inducing protective immunity [33, 34]. In addition, the choice of adjuvant may be crucial for generating strong and balanced immune responses against these respiratory pathogens.

**Fig 1:**
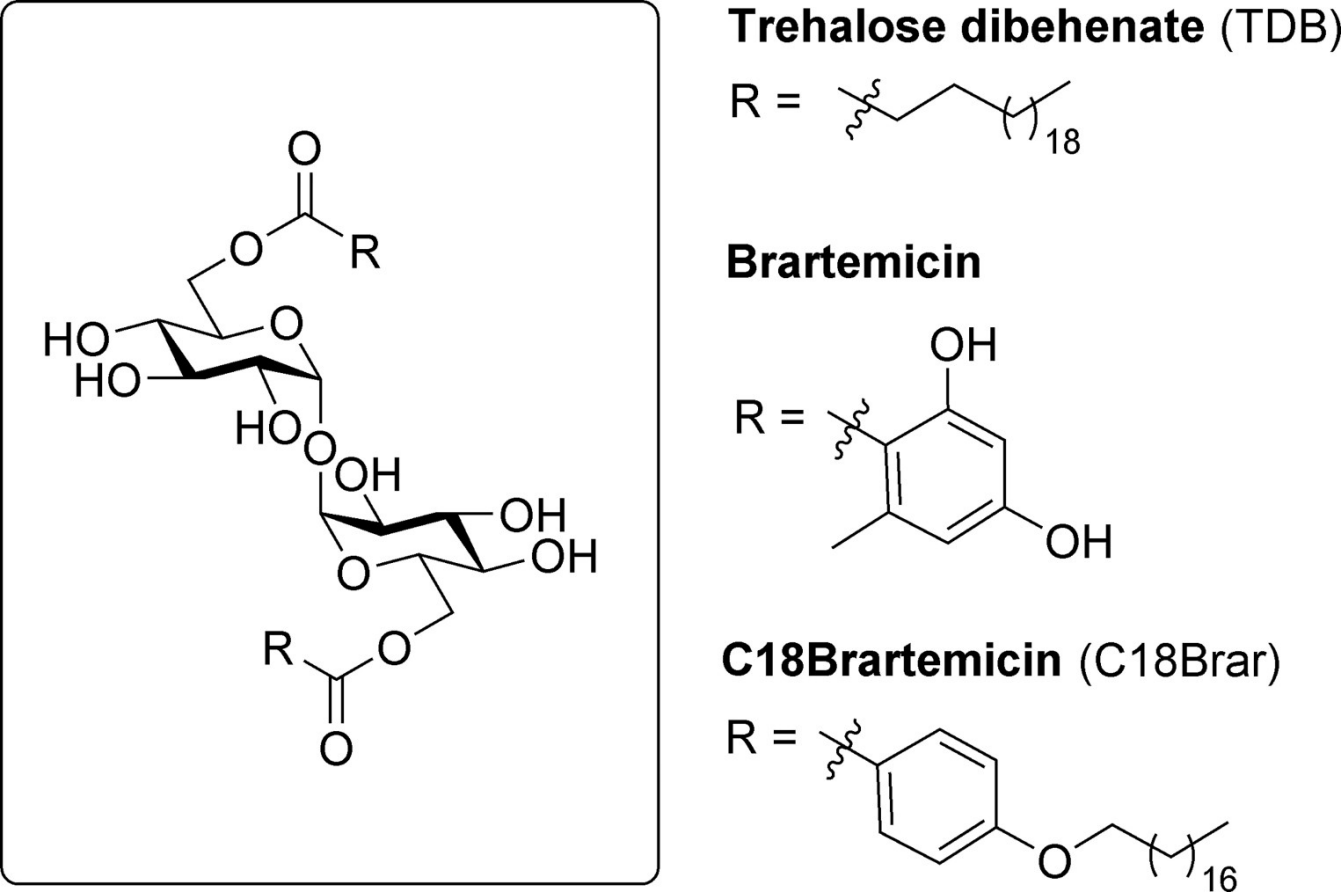
Representative trehalose glycolipids, trehalose dibehenate (TDB), the natural product brartemicin, and a lipophilic derivative thereof, C18Brartemicin (C18Brar). TDB and C18Brar are potent ligands for the C-Type lectin, Mincle.

Pathogen-associated molecular patterns (PAMPs) have been widely used as vaccine adjuvants to enhance immune responses [35]. PAMPs specifically bind to pattern-recognition receptors (PRRs) on innate immune cells, thereby ensuring activation of specific immune responses in cell- and PRR-dependent manners [36]. The specific binding of PAMPs to their respective PRRs leads to enhanced cell-mediated immunity by improving the presentation of antigens to antigen-presenting cells (APCs) [35]. Various agonists of Toll-like receptor (TLR) PRRs have been described as vaccine adjuvants [37], while more recently, targeting the macrophage inducible C-type lectin (Mincle, Clec4e, or Clecsf9), a PRR on the surface of macrophages and dendritic cells (DCs), has shown particular promise as a means to augment vaccine efficacy [38–40]. Mincle is activated by several PAMPs including the *Mycobacterium tuberculosis* cell wall glycolipid trehalose dimycolate (TDM) [41] and its C22 synthetic counterpart, trehalose dibehenate (TDB, Fig 1) [42], as well as α-mannosyl residues found on Malassezia fungi [43]. To date, there have only been limited studies examining the potential of Mincle adjuvants to augment immune responses for veterinary vaccine antigens [44], with studies into the use of TDB and amide-derivatives of TDB being the first to demonstrate the potential of these ligands in *in vivo* veterinary models [39, 45, 46].

Recent studies have demonstrated that C18Brar (Fig 1), a lipophilic analogue of the trehalose diester brartemicin, is a potent Mincle agonist that activates APCs in a Mincle-dependent manner [38, 47]. We investigated the structure-activity-relationships of lipophilic brartemicin derivatives [38, 47, 48], along with other trehalose glycolipids, [49, 50] and observed that incorporation of the aromatic group of the brartemicin scaffold along with the lipophilic chain leads to enhanced antigen-specific humoral- and cell-mediated responses in mice, as compared to TDB when using OVA as a model antigen [38, 47]. The potential of C18Brar to act as an adjuvant for veterinary vaccines has not been investigated. Mincle is highly conserved across mammalian species, and the ease of synthesis of C18Brar makes it an ideal candidate adjuvant for veterinary vaccines. In this study, we evaluated the immune responses of sheep to a vaccine containing a mixture of inactivated *M*. *haemolytica* and *M*. *ovipneumoniae* whole cells formulated with C18Brar. The immune responses were compared to those induced by formulations containing the commercially available adjuvants Quil-A [51], Alhydrogel [52], and Emulsigen-D [53].

## Materials and methods

### Animals

Romney cross lambs, 6-months of age, were sourced from a commercial farm and used for the comparative adjuvant study (Table 1). These animals were selected from a larger group of lambs (*n* = 100) to eliminate animals with high pre-existing serum antibody responses to *M*. *haemolytica* and *M*. *ovipnuemoniae* antigens. Animals with ELISA OD_450_ values < 0.3 at a 1:50 dilution of serum, were used for the vaccination study. All animals were grazed on pasture with water *ad libitum*. Animal ethics approval for the trial was obtained from the AgResearch Grassland’s Animal Ethics Committee, Palmerston North, New Zealand.

**Table 1:** Vaccine groups and formulation of vaccines.

	Formulation of vaccine	
Animal group	Bacterial Antigens	Adjuvants
1	+	-
2	+	Quil-A
3	+	Emulsigen-D
4	+	Alhydrogel + Quil-A
5	+	C18Brar

### Bacteria

*M*. *haemolytica* X387 was provided by MSD Animal Health (Upper Hutt, New Zealand). This strain hyper-produces LKT and was inactivated with formalin by the manufacturer and is an antigen component of the commercial vaccine Multine MH^®^ + IPR (MSD Animal Health). The culture was stored at 4°C until used. The bacterial cells were blended by vortexing with the adjuvant components at a final concentration of 50% *v/v*.

Three New Zealand isolates of *M*. *ovipneumoniae* #16, 90, and 103, representative of the three dominant genetic types prevalent in NZ sheep [14], were cultured in Frey’s medium [54]. Cultures were inactivated by treatment with 5% H_2_O_2_ for 2 h at room temperature using a previously described method [55], washed twice with 10 mM, pH 7.3 phosphate-buffered saline (PBS) and resuspended in PBS at a protein concentration of 9 mg/mL. Protein concentration was measured using the Bradford assay according to the manufacturer’s instruction (Thermo Fisher Scientific, NZ). Each vaccine dose contained 0.6 mg of total protein from each isolate.

### Adjuvants and formulation of vaccines

Vaccines were prepared by combining bacterial antigens with adjuvants in a vaccine dose volume of 2.5 mL. Quil-A^®^, a saponin adjuvant, was obtained from InvivoGen (InvivoGen USA, San Diego, CA, USA). A stock solution (5 mg/mL) of Quil-A was prepared in distilled water and filter-sterilised through a 0.22 μm filter. Each vaccine dose contained 1.5 mg of Quil-A. Antigens were formulated with a mixture of aluminium hydroxide gel (Alhydrogel, InvivoGen) and Quil-A. Alhydrogel and Quil-A were mixed with antigens at a final concentration of 5 mg and 0.5 mg per vaccine dose, respectively. Emulsigen^®^-D is an oil-in-water dual adjuvant emulsion containing dimethyldioctadecyl ammonium bromide (DDA) in nanoparticles (MVP Adjuvants®, Phibro Animal Health Corporation, Teaneck, NJ, USA). Emulsigen^®^-D was combined with the protein antigens in the vaccine at a final concentration of 20% *v*/*v*.

**Fig 2:**
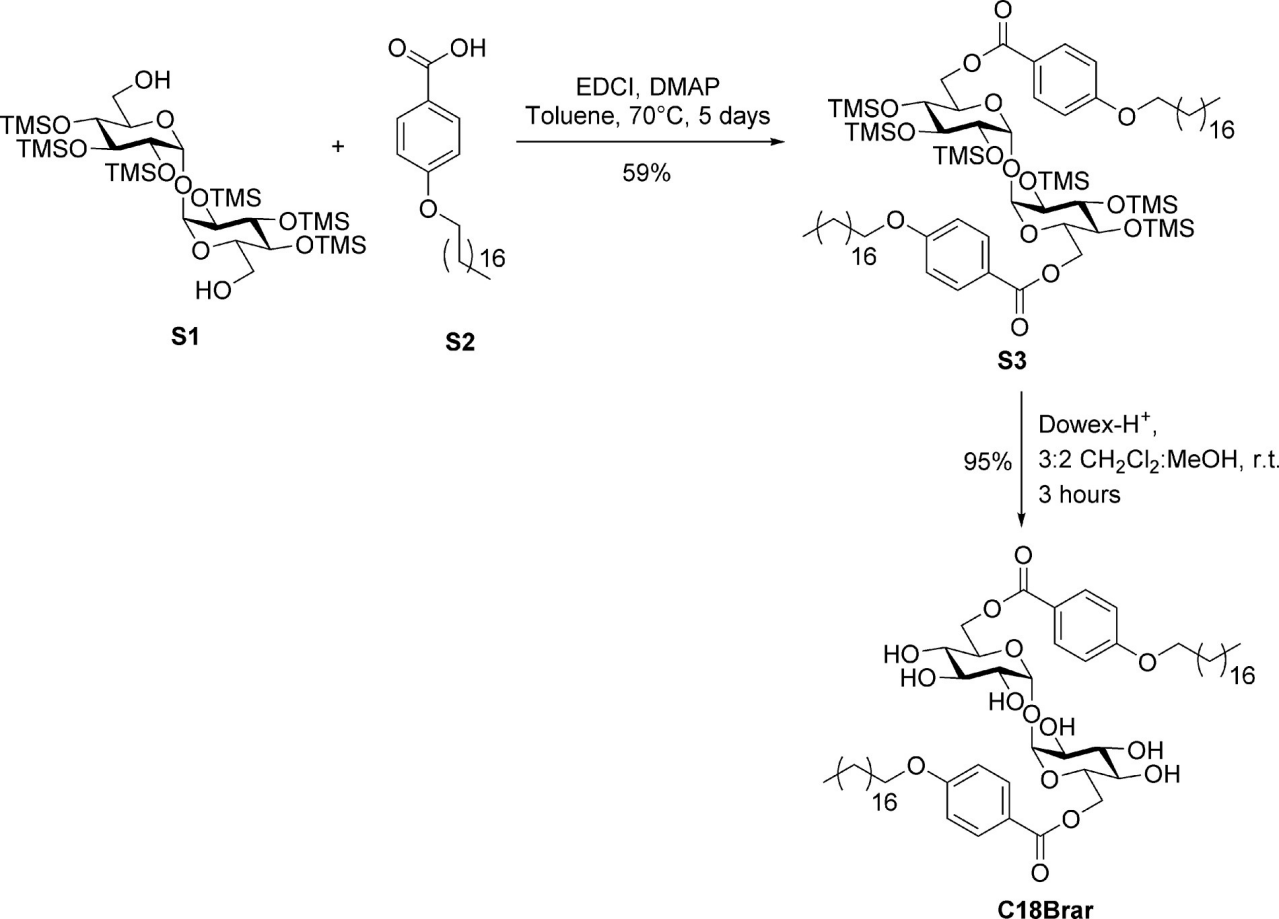
Illustration showing synthesis of C18Brar. To prepare C18Brar, 2,2’,3,3’,4,4’-hexa-*O*-trimethylsilyl-α,α’-D-trehalose **S1** [56] and 4-octadecyloxy-benzoic acid **S2** [38] were treated with *N*-ethyl-*N’*-(3-dimethyaminopropyl)carbodiimide (EDCI) and 4-dimethylaminopyridine (DMAP), to form the diester **S3** in an 59% yield. The trimethylsilyl ether protecting groups were subsequently removed with acidic Dowex to give C18Brar in 95% yield. Characterization data was consistent with that previously reported [38].

A stepwise illustration showing the synthesis of the trehalose adjuvant C18Brar (C18-alkylated brartemicin analogue, [56]) is given (Fig 2). The detailed procedure for C18Brar preparation is provided in S1 File. The purity of C18Brar was determined by ^1^H NMR and HRMS, and preparations were determined to be endotoxin free (≤ 0.1 EU/mL) by using the limulus amoebocyte (LAL) chromogenic assay. To assess the immunological effect of C18Brar alone, C18Brar was prepared at 3 mg/mL in 9:1:40 mineral oil:Tween-80:PBS (*v/v/v*), rather than in a liposomal formulation containing immunologically active DDA, which is often used in vaccines containing TDB. To prepare the emulsion, C18Brar was crushed to a fine powder then dissolved in mineral oil by mixing (vortexing). Tween-80, followed by PBS were added and the resulting emulsion mixed by vortexing and sonication for 30 min and stored at 4°C prior to use. For vaccine formulation, the preparation was diluted 1:1 with bacterial antigens in PBS by gentle mixing to form a homogeneous emulsion 1 day prior to vaccination. Each vaccine dose contained 3.75 mg of adjuvant.

### Vaccination and sampling of animals

Sixty sheep were allocated randomly to 5 groups each with 12 animals (Table 1). Vaccines were administered by the intramuscular route in the anterior region of the neck. Animals were re-vaccinated with the same vaccine 3 weeks after the first vaccination. Blood samples were collected at week 0 (before vaccination) and 3, 6, 9, 13, and 34 weeks post-vaccination and sera was prepared by centrifugation at 2,000 × g for 10 min at room temperature and stored at –20°C until further analysis. Additionally, blood samples were collected in heparinised blood collection tubes at weeks 0 and 6 for the determination of T-cell responses.

### Monitoring of vaccination sites

The vaccination sites for all animals were monitored daily for three days following the first and second vaccinations. Monitoring was also performed routinely on a weekly basis for up to 4 weeks after the second vaccination.

The vaccination area was monitored for lump formation with signs of either soft or hard tissue, evidence of wool break, redness, pain on palpation or scab. The lumps were measured in length, breadth and height using a small ruler. Each lump was assessed and scored as: 0, no oedema–flat skin; 1, very slight oedema–barely noticeable; 2, slightly raised oedema with well-defined edging, <1 cm in size; 3, raised oedema with well-defined edging and > 1 cm in height, < 6 cm in diameter; 4, moderate oedema and raised > 1 cm in height and > 6 cm in diameter; 5, burst abscess. If an abscess formed and burst at the vaccination site, the abscess was treated with Tetravet antibiotic spray (Bayer, Australia).

### Measurement of antibodies using ELISA

An ELISA assay was used to measure *M*. *ovipneumoniae* and *M*. *haemolytica* antigen-specific IgG responses in serum as described previously [39]. Briefly, MaxiSorp high protein-binding capacity 96 well ELISA plates (Nunc™; Thermo Fisher Scientific) were coated overnight at 4°C with 50 μL/well of *M*. *haemolytica* whole cell antigens (14.3 μg/mL protein) in PBS or *M*. *ovipneumoniae* whole cell antigens in 50 mM sodium carbonate buffer, pH 9.6. The bacterial antigens of the inactivated cultures used to formulate the vaccines were washed with PBS before measuring protein concentration and then were used for coating the plates. The following day, the plates were washed with PBS + Tween-20 (0.5%) (PBST) and blocked for 1 h at room temperature with 100 μL/well of blocking buffer (PBS containing 1% (*w/v*) casein). After incubation, the plates were washed with PBST, and 2-fold serial dilutions of sera (range 1:200–1:204,800 diluted in blocking buffer) were added (50 μL/well). The pre-vaccination and post-vaccination sera of an animal were tested on the same plate. The plates were incubated for 1 h at room temperature, washed with PBST, then incubated for 1 h at room temperature with either HRP-conjugated donkey anti-sheep IgG (BioRad, CA, USA) diluted at 1:6,000, rabbit anti-sheep IgA (Bethyl Laboratories, TX, USA) at 1:3,000, or rabbit anti-sheep IgM (BioRad, CA, USA) at 1:5,000 in blocking buffer (50 μL/well). Following washing with PBST, 50 μL/well of 3,3′,5,5′-Tetramethylbenzidine (TMB) substrate (BD Biosciences) was added, and the plates incubated for 20 min at room temperature in the dark. The reactions were stopped with the addition of 50 μL/well of 0.5 M H_2_SO_4_ and the absorbance read at 450 nm using a microplate reader (VERSAmax, Molecular Devices). For each animal, the antibody titre of each post-vaccination serum was calculated from the reciprocal of the highest dilution showing an OD_450_ value greater than the OD_450_ value of a 1:200 dilution of pre-vaccination serum.

### Production of leukotoxin (LKT) and measurement of LKT-specific antibodies

Leukotoxin (LKT) was produced and LKT-specific antibodies were measured by ELISA according to previously described methods [57]. Briefly, *M*. *haemolytica* serotype 1 was cultured overnight at 37°C on Colombia sheep blood agar plates (Fort Richard, Auckland, NZ) and used to inoculate 500 ml RPMI 1640 medium (Invitrogen, ThermoFisher Scientific) supplemented with 0.5% bovine serum albumin (Thermo Fisher Scientific) in a 2 L flask. The culture was incubated at 37°C, on a rocking platform (70 rpm) until the OD600 reached 0.8–1.0. Bacterial cells were pelleted by centrifugation at 12,785 × g for 30 min at 4°C and the LKT was purified from the culture supernatant by fractional ammonium sulfate precipitation (0–60% saturation). SDS-PAGE electrophoresis of the preparation showed a dominant protein band at the expected molecular size (105 Kda) and the identity of LKT was confirmed by mass spectrometry (data not shown).

MaxiSorp high protein-binding capacity 96 well ELISA plates (Nunc™; Thermo Fisher Scientific) were coated overnight at 4°C with 50 μL/well of LKT antigens diluted in carbonate-bicarbonate buffer at 0.5 μg/mL concentration. The remainder of the steps were the same as those described for IgG, except that the HRP-conjugated donkey anti-sheep IgG (BioRad, CA, USA) was used at 1:3,000 dilution. For each animal, the antibody titre of each post-vaccination serum was calculated from the reciprocal of the highest dilution showing an OD_450_ value greater than the OD_450_ value of a 1:400 dilution of pre-vaccination serum.

### Measurement of cellular immune responses

For the measurement of IFN-γ and IL-17A responses, blood was collected in heparinised blood tubes at both pre-vaccination (week 0) and post-vaccination (week 6). Heparinised whole blood (1 mL) was diluted 1:1 with RPMI 1640 medium (Invitrogen, Thermo Fisher scientific) containing 5% foetal bovine serum (Invitrogen) and incubated in 24-well plates (total of 2 mL per well) with *M*. *haemolytica* or *M*. *ovipneumoniae* whole cell antigens (same antigens as used for formulating the vaccines, except the culture of *M*. *haemolytica* was washed with PBS prior to use to remove any traces of formalin). Prior to the trial, the concentration of antigens for maximum stimulation of cytokine release was optimised. For the determination of cytokine release at weeks 0 and 6, *M*. *haemolytica* and *M*. *ovipnuemoniae* antigens were used at a final concentration of 14 and 10 μg/mL total cell protein, respectively. As a positive control, pokeweed mitogen (PWM) was added to a well at a final concentration of 2.5 μg/mL), while PBS was added to another well as a negative control.

Following incubation at 37°C, 5% CO_2_ for 40 h, the cultures were centrifuged at 400 × g for 10 min and plasma was carefully removed and stored at –20°C until assayed for the release of cytokines. The concentration of IFN-γ in the plasma was measured by ELISA using a Bovigam ELISA kit (Prionics, Thermo Fisher Scientific), which measures IFN-γ in cattle, sheep, goats, and other bovidae. An ovine IFN-γ standard (Kingfisher Biotech, St. Paul, USA) was titrated and the concentration of IFN-γ was calculated from the standard curve.

ELISA protocols for ovine-specific IL-17A were developed and optimised in-house using capture, detection antibodies and recombinant ovine IL-17A as standards (Kingfisher Biotech) according to the manufacturer’s instructions. Briefly, MaxiSorp high protein-binding capacity 96 well ELISA plates (Nunc™) were coated overnight at room temperature with 50 μL/well of capture antibody (2 μg/mL protein) in PBS. The plates were washed with PBST and blocked for 1 h with 100 μL/well of blocking buffer (PBS containing 4% (*w*/*v*) BSA) at 37°C with shaking. Following blocking, the plates were washed again with PBST. Ovine IL-17A standards and undiluted plasma samples (50 μL/well) were added to the plates and the plates were incubated for 1 h at 37°C. Following the incubation, the plates were washed with PBST and incubated for 1 h at 37°C with biotin-conjugated detection antibody (Kingfisher Biotech) diluted at 1:4,000 in blocking buffer (50 μL/well). After incubation, the plates were washed with PBST, and the plates were incubated for 30 min at 37°C with streptavidin diluted at 1:500 in blocking buffer (50 μL/well). Following incubation, the plates were washed with PBST, and 50 μL/well of TMB substrate (BD Biosciences) added, and the plates incubated 20 min at room temperature in the dark. The reactions were stopped by addition of 0.5 M H_2_SO_4_ (50 μL/well) and absorbance read at 450 nm using a microplate reader (VERSAmax, Molecular Devices). The concentration of IL-17A for each sample was calculated from the standard curve.

### Measurement of gene expression by Nanostring nCounter

Diluted blood cultures were incubated with *M*. *haemolytica* and *M*. *ovipneunoniae* antigens as performed for measurement of IFN-γ and IL-17A. Following incubation for 24 h and removal of plasma, 1 mL of TRI Reagent® LS (Invitrogen) was added to the cells and the homogenates stored at –80°C until the RNA was isolated. Total RNA was isolated using a Direct-zol RNA Kit (Zymo Research, Irvine, CA, USA) according to the manufacturer’s instructions. The quality and quantity of RNA was measured using a NanoDrop spectrophotometer (Thermo Fisher Scientific). Gene expression analysis was performed using Nanostring nCounter technology (Nanostring Technologies Inc., Seattle, WA) and some modifications to a previously described method [58]. Sequence-specific capture and reporter probes for each gene of interest are used in the amplification-free system. The capture probe is coupled to biotin as an affinity tag, while the reporter probe is coupled to a color-coded tag. The probes specifically hybridize to the target gene sequences and generate the unique colour code by the ordered fluorescent tags on the reporter probe resulting in identification of the target gene. The level of expression is measured by counting the number of codes for each mRNA using digital imaging. This allows for the analysis of multiple genes from the same sample (multiplexing) using a customised set of probes with distinct bar codes, called a ProbeSet.

A titration was performed using sheep-specific ProbeSets (S1 Data) and a PlexSet-24 titration kit according to the manufacturer’s instructions (Nanostring Technologies). A total of 1.2 μg of purified RNA was used to measure the expression of various immune response genes (S1 Table) using a PlexSet-24 (Nanostring Technologies) according to the manufacturer’s instructions. RNA samples were hybridized with the ProbeSets according to the manufacturer’s instructions (nCounter PlexSet Reagents User Manual; Nanostring MAN-10040-05). Briefly, samples were hybridized by adding 8 μL of MasterMix and 7 μL of RNA to 0.2 mL PCR tubes in a 12-tube strips and immediately placed at 67°C for 24 h. After hybridization, samples were transferred to the nCounter Prep Station, which robotically removed the excess probes and aligned as well as immobilized the probe-target complexes onto the nCounter cartridge. The processed cartridges were placed in the nCounter Digital Analyzer to count the codes on the surface of the cartridge for each target mRNA. The mRNA counts were generated in a tabulated form and were retrieved from the analyzer as raw data (Reporter Code Count, RCC) files.

The RCC files were imported into nSolver Analysis Software version 4 (https://www.nanostring.com/products/analysis-software/nsolver) for analysis. The software performed quality control routine to flag the samples for exclusion according to the following parameters: fields of view registration < 75%; binding density outside the 0.05 to 2.25 range; positive control linearity: positive control R^2^ value < 0.95; and positive control limit of detection: 0.5 fM positive control ≤ 2 SD above the mean of the negative controls. All samples used for statistical analysis passed the quality control routine.

Background subtraction was performed by subtracting the geometric mean of 8 internal negative controls from each sample. Positive control normalization was performed using the geometric mean of 6 internal positive controls to compute the normalization factor. The normalization factor of all samples was inside the 0.65 to 1.67 range.

Reference gene normalization was performed using the geometric mean of counts for the three reference genes included in the ProbeSet (S1 Table). The average of these geometric means across all lanes was used as the reference against which each lane is normalized. A normalization factor was then calculated for each of the lanes based on the geometric mean of counts for the reference genes in each lane relative to the average geometric mean of counts for the reference genes across all lanes. This normalization factor was then used to adjust the counts for each gene target and controls in the associated lane. The normalization factor of all samples was inside the 0.3 to 23 range.

### Statistical analysis

Analysis of antigen-specific antibody levels and gene expression were based on mixed effects model using package ‘nlme’ [59] in R version 4.1.1 [60]. The immunoglobulin stability experiment was a randomised block design with two treatments “group” and “time”, a mixed effects model with fixed effects “group”, “time” and their interaction, and a random effect “animal” was used in the analysis. For matching the assumption of normality, either a square root or log_e_ transformation was applied to the response variable. Unit values of antibody responses in sheep were log_2_-transformed prior to statistical analysis. To detect groups of different adjuvants and at different weeks and their interactions, day 0 data (before vaccination) were treated as covariate, groups and weeks and their interactions as fixed effects, and individual animals as random effects. Individual comparisons were made using a post-hoc multiple comparison test [61]. The *P* values from the test were adjusted by the “BH” method to control for false discovery rate [62]. The level of significance was set at a *P* value of < 0.05.

For the IFN-γ and IL-17A responses a one-way ANOVA was applied to log-transformed values. *P* values < 0.05 were considered statistically significant.

## Results

### Vaccination of animals

Sheep were vaccinated twice at a 3-week interval and the vaccination sites were observed after the administration of each vaccine. A proportion of animals in each group produced a lump at the first or second vaccination site with a score 3 or higher, including some that were vaccinated with inactivated *M*. *haemolytica* and *M*. *ovipneumoniae* whole cells formulated with C18Brar (Table 2). These lumps were considered minor in nature and only one animal developed an abscess which burst and was treated with antibiotic spray. The other adjuvants tested also caused lumps in a proportion of animals, most notably with Emulsigen-D. All observed lumps were transient and disappeared during the time course of the trial. The few lumps that developed into abscesses healed and did not cause discomfort to the animals and there were no animal welfare concerns.

**Table 2:** Proportion of animals showing vaccination site reactions (lumps).

Group	Proportion of animals producing an observable reaction at each vaccination site with a score of 3 or 4 or [5]	
	1^st^ vaccination site	2^nd^ vaccination site
**Ags alone**	1/12 [0/12]	1/12 [0/12]
**Quil-A**	7/12 [0/12]	6/12 [0/12]
**Emulsigen-D**	10/12 [3/12]	10/12 [3/12]
**Alhydrogel + QuilA**	2/12 [0/12]	7/12 [0/12]
**C18Brar**	11/12 [1/12^a^]	9/12[1/12^a^]

^a^ Same animal for both 1^st^ and 2^nd^ vaccination sites

Scores: 3, raised oedema with well-defined edging and > 1 cm in height, < 6 cm in diameter; 4, moderate oedema, raised > 1 cm in height and > 6 cm in diameter; 5, burst abscess.

### C18Brar promotes IgG antibody responses to *M*. *haemolytica* and *M*. *ovipneumoniae* antigens

The ability of C18Brar to induce IgG antibody responses to the antigens present in inactivated *M*. *haemolytica* and *M*. *ovipneumoniae* whole cells was evaluated and compared with three other adjuvant systems, Quil-A, Emulsigen-D, and a mixture of Alhydrogel and Quil-A. A combination of Alhydrogel and Quil-A was used as we had observed that aluminium hydroxide (alum) alone induced weaker antibody responses to *M*. *haemolytica* than Quil-A (data not shown). The addition of a small amount of Quil-A to alum adjuvants was suggested to improve the efficacy of an alum-based adjuvant system (MSD Animal Health, personal communication).

**Fig 3:**
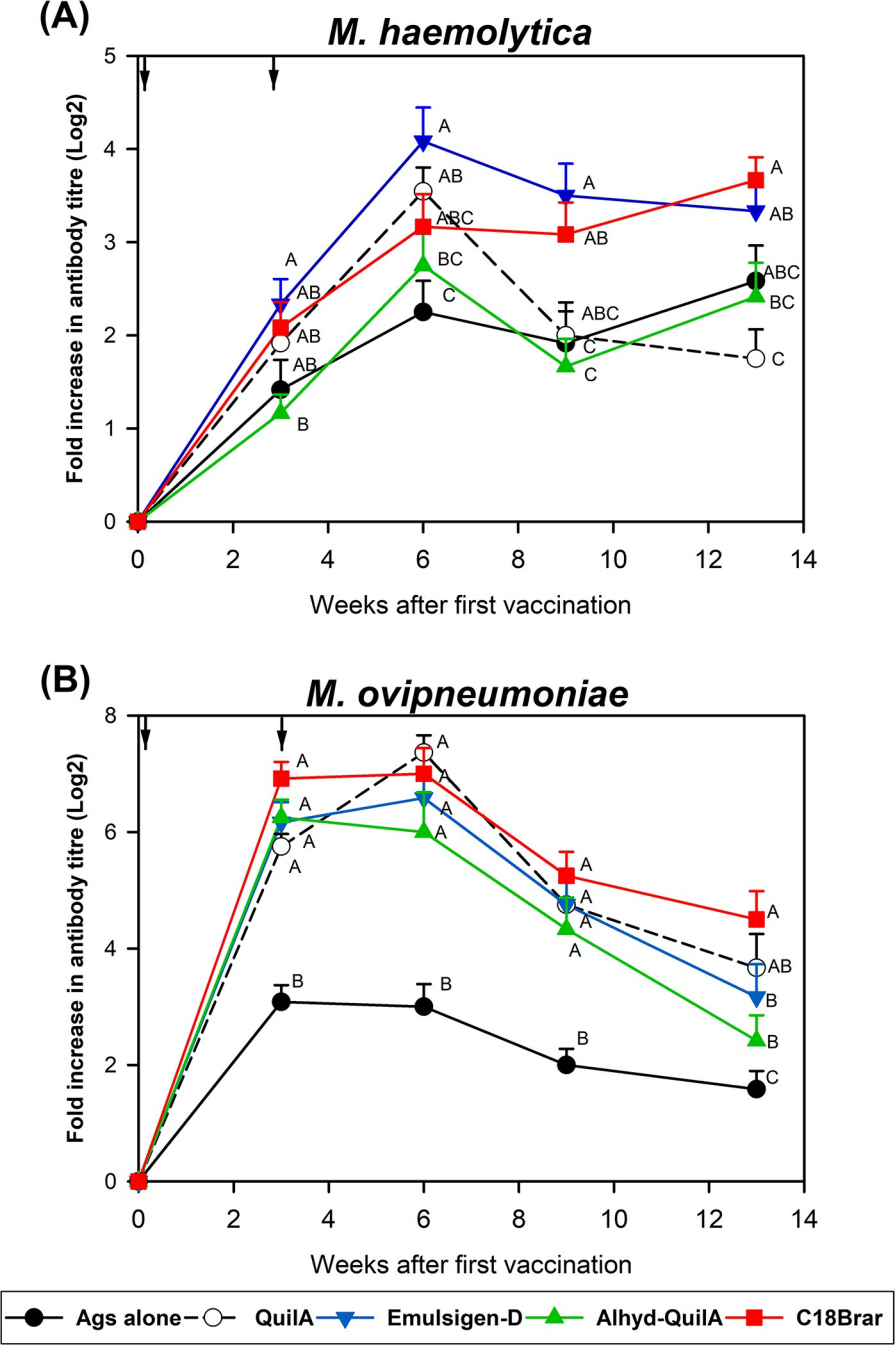
IgG antibody responses to *M*. *haemolytica* and *M*. *ovipneumoniae* formulated in different adjuvants. Mean (+SE) serum IgG antibody responses to *M*. *haemolytica* and *M*. *ovipneumoniae* in animals vaccinated with a mixture of *M*. *haemolytica* and *M*. *ovipneumoniae* whole cell antigens formulated with different adjuvants or given antigens (Ags) alone at week 0 and 3 (timing of vaccinations shown by arrows). Antibody responses were measured in serum samples collected at weeks 0, 3, 6, 9, and 13 using ELISA. Different alphabetical letters indicate significant differences (*P* < 0.05) between the groups, while the same letter indicates no significant difference between groups.

Animals vaccinated with *M*. *haemolytica* and *M*. *ovipneumoniae* antigens formulated with C18Brar adjuvant produced higher antibody titres to *M*. *haemolytica* antigens at weeks 9–13 and to *M*. *ovipneumoniae* at weeks 3–13 compared to the control antigens alone group (*P* < 0.05, Fig 3A & 3B). Animals vaccinated with antigens formulated with Quil-A adjuvant produced higher antibody titres to *M*. *haemolytica* antigens at week 6 and to *M*. *ovipneumoniae* antigens at all time points compared with animals vaccinated with antigens alone (*P* < 0.05, Fig 3A & 3B). The Emulsigen-D adjuvant produced higher antibody titres to *M*. *haemolytica* compared with the antigen alone group at weeks 6, 9, and 13, and had higher antibody responses to *M*. *ovipneumoniae* at weeks 3–13 (*P* < 0.05, Fig 3A & 3B). The combination of Alhydrogel and Quil-A adjuvants produced higher *M*. *ovipneumoniae*-specific antibody responses (*P* < 0.05, Fig 3A & 3B) at weeks 3–13 compared to the antigen alone group but no differences were observed between the two groups for responses to *M*. *haemolytica* (Fig 3A). In addition, some differences were also observed between different adjuvants. For example, *M*. *haemolytica* and *M*. *ovipneumoniae* antigens formulated with C18Brar and Emulsigen-D induced stronger *M*. *haemolytica*-specific antibody titres compared to the levels induced by QuilA and Alhydrogel + QuilA adjuvants at weeks 9 and 13. Similarly, the antigens formulated with C18Brar led to stronger *M*. *ovipneumoniae*-specific antibody titres compared to Emulsigen-D and Alhydrogel + QuilA adjuvants at week 13 (Fig 3B).

In addition, IgM and IgA antibody responses were also measured in the vaccinated animals. Only weak to moderate IgM antibody responses were measured in a proportion of animals vaccinated with *M*. *haemolytica* and *M*. *ovipneumoniae* whole cells antigens formulated with the various adjuvants and there were no significant differences between groups (S5 Fig in S1 File). No IgA responses were observed in the vaccinated animals (data not shown).

### Antibody responses to *M*. *ovipneumoniae* antigen formulated with C18Brar adjuvant persist long-term

**Fig 4:**
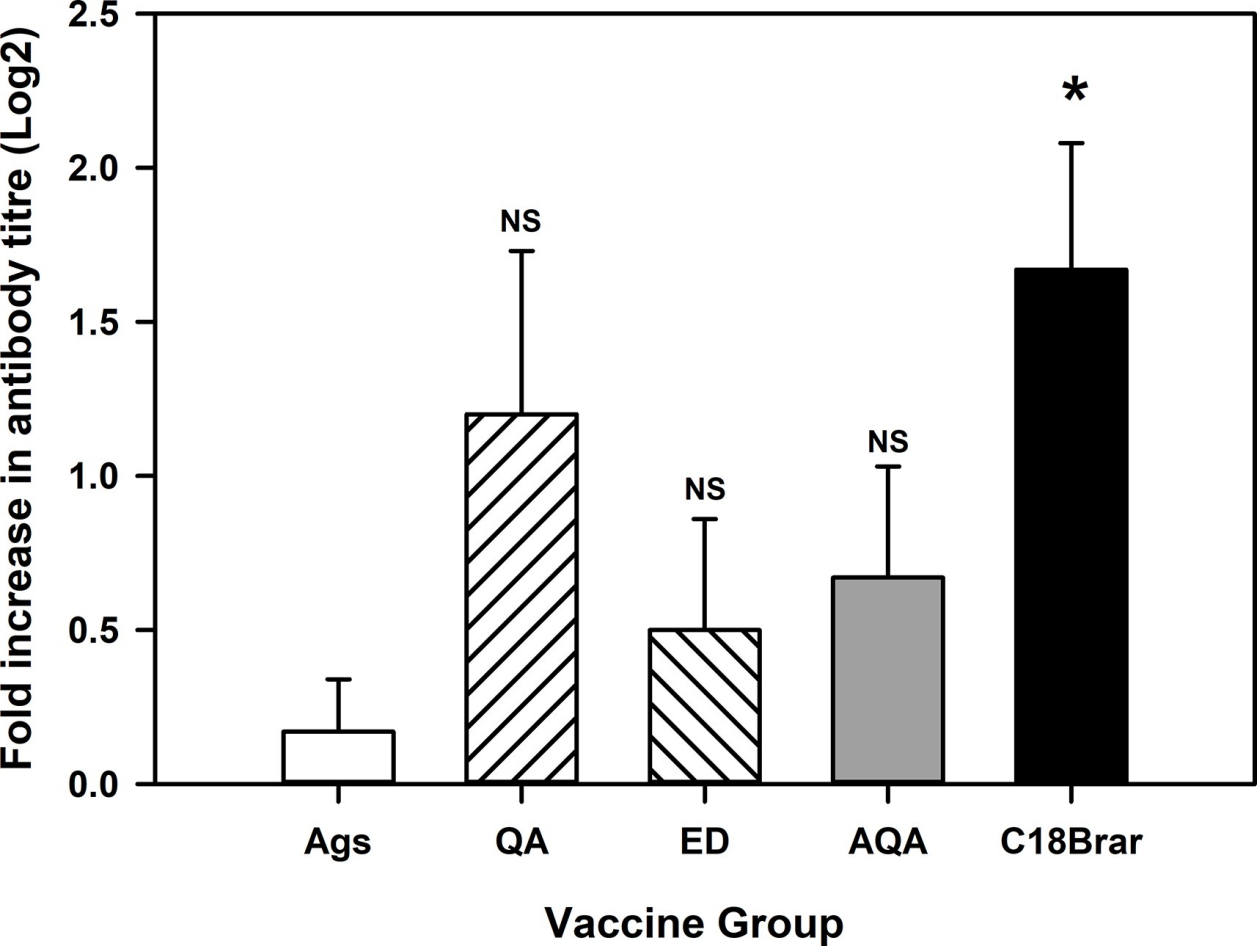
IgG antibody responses to *M*. *ovipneumoniae* antigen promoted by C18Brar adjuvant persist long-term. Mean (+SE) serum IgG antibody responses to *M*. *ovipneumoniae* at 34 weeks after initial vaccination in animals vaccinated with either Antigens alone (Ags), antigens formulated with Quil-A (QA), Emulsigen-D (ED), Aldhydrogel + QuilA (AGA), or C18Brar. Antibody responses were measured in serum samples using ELISA. Significant differences are represented by * *P* = < 0.05 in antibody responses compared to Ags alone group. NS = non-significant.

The level of *M*. *ovipneumoniae* IgG specific antibody responses were particularly encouraging when using several of the adjuvants compared to antigen alone. To determine the longevity of the antibody responses, levels of serum antibodies were remeasured at week 34 (31 weeks after the second vaccination). Although the antibody titres had declined further from the levels recorded at week 13, IgG antibody titres against *M*. *ovipneumoniae* antigens in animals administered C18Brar were higher than the control animals (*P* < 0.05), and significantly higher than those observed following the administration of Emulsigen-D (Fig 4). Responses to *M*. *haemolytica* at week 34 were minimal, with no differences observed between groups (data not shown).

### C18Brar promotes antibody responses to *M*. *haemolytica* leukotoxin

**Fig 5:**
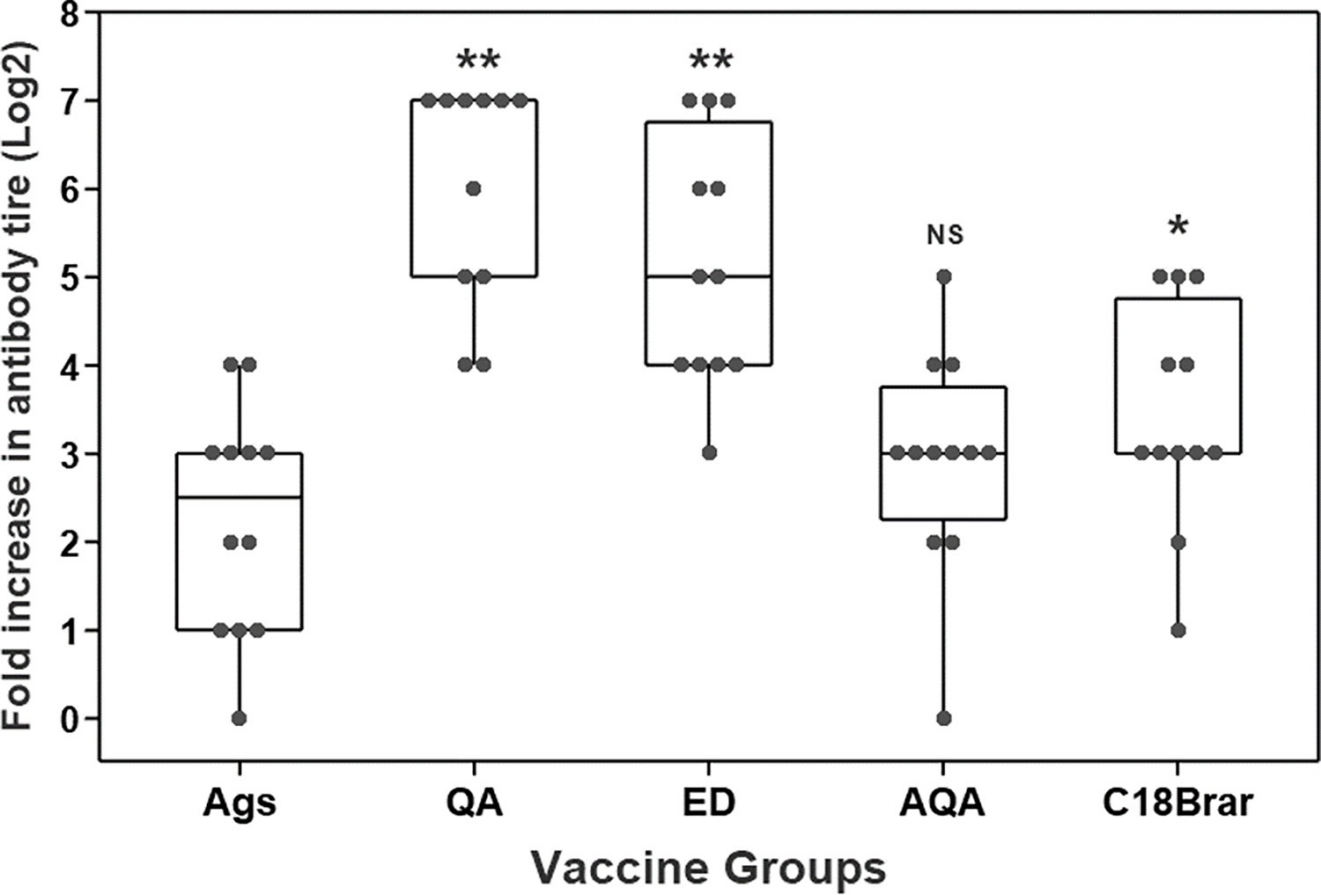
Antibody responses to leukotoxin in the vaccinated animals. Serum antibody responses to *M*. *haemolytica* leukotoxin in animals vaccinated with a mixture of *M*. *haemolytica* and *M*. *ovipneumoniae* whole cell antigens formulated with different adjuvants or given antigens (Ags) alone. Antibody responses were measured in serum samples collected at weeks 0 and 6 using ELISA. An individual animal is represented by ‘●’ and significant differences are represented by * = < 0.05, ** = < 0.01 compared to Ags alone group. NS = non-significant.

The ability of the adjuvants to induce antibody responses to LKT was also evaluated in the vaccinated animals. Animals vaccinated with antigens formulated with C18Brar produced significantly higher (*P* < 0.05) LKT-specific antibody responses compared to the animals vaccinated with antigens alone at week 6 (Fig 5). Emulsigen-D and QuilA also stimulated significantly higher (*P* < 0.01) anti-LKT antibodies compared to control animals (Fig 5).

### C18Brar promotes IL-17A responses to *M*. *haemolytica* and *M*. *ovipneumoniae* whole cell antigens

**Fig 6:**
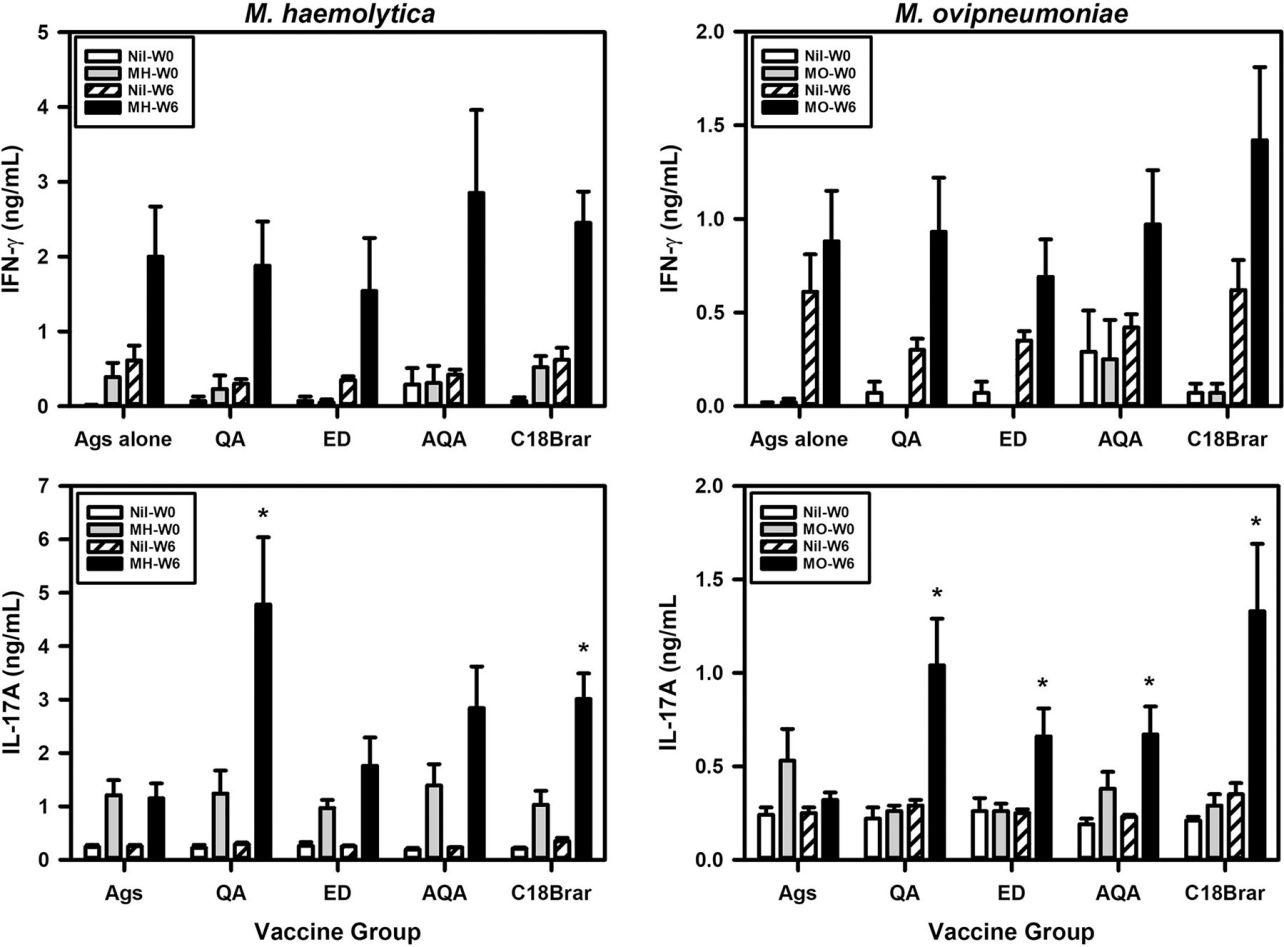
Cytokine responses to *M*. *haemolytica* and *M*. *ovipneumoniae* whole cell antigens in a whole blood stimulation assay. Mean (+SE) IFN-γ, and IL-17A responses to PBS (Nil), *M*. *haemolytica* (MH) and *M*. *ovipneumoniae* (MO) whole cell antigens at week 0 (W0) and week 6 (W6). Blood samples from the animals vaccinated with Antigen alone (Ags), Quil-A (QA), Emulsigen-D (ED), Alhydrogel + QuilA (AQA), and C18Brar were stimulated for 40 h with *M*. *haemolytica* and *M*. *ovipneumoniae* whole cell antigens. Cytokine release was measured by ELISA and the reported values were calculated from the values for antigen stimulation where PBS was used as a control. For IL-17A, significant differences are represented by * = *P* < 0.05 compared to Ags alone group at week 6, while no significant differences between groups were observed for IFN-γ levels.

The ability of the adjuvants to induce cellular immune responses in the vaccinated animals was evaluated by measuring the release of IFN-γ and IL-17A from antigen-stimulated whole blood cultures. There were no significant differences in IFN-γ responses at week 6 between the groups (Fig 6), which was due, at least in part, to the high variability in responses between individual animals within groups. C18Brar and Quil-A elicited higher levels of IL-17A production in whole blood cultures stimulated with *M*. *haemolytica* antigens compared to levels in the antigen alone group (*P* < 0.05, Fig 6). All adjuvants promoted IL-17A responses to *M*. *ovipneumoniae* antigens, with IL-17A responses in all four groups of animals given adjuvants being higher than those from animals vaccinated with antigens alone (control group) (*P* < 0.05, Fig 6). Minimal levels of cytokine release were measured in the *in vitro* stimulated blood cultures at week 0. The blood cultures responded to the positive control PWM as expected with mean (± SE) values across all animals for IL-17A of 7.6 ± 0.7 and 6.1 ± 0.5 ng/mL at weeks 0 and 6, respectively.

### Expression of immune response genes

**Fig 7:**
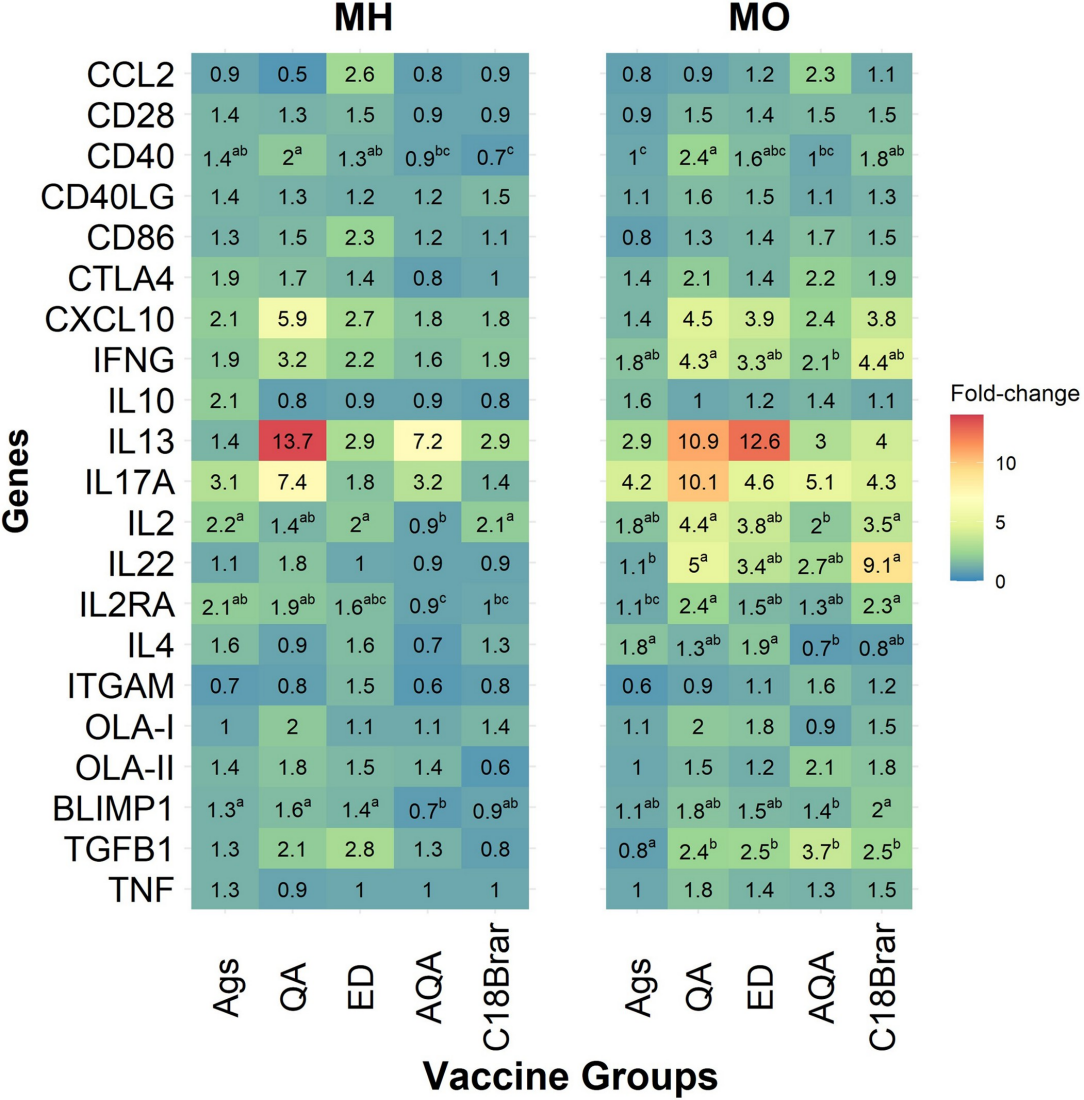
Relative expression of immune responsive genes. Whole blood samples were collected from animals before and after vaccination of animals with either a mixture of *M*. *haemolytica* and *M*. *ovipneumoniae* whole cell antigens alone, or antigens formulated with Quil-A (QA), Emulsigen-D (ED), Alhydrogel + Quil-A (AQA), or C18Brar adjuvants. Blood cultures were stimulated *in vitro* with either PBS, *M*. *haemolytica* (MH), or *M*. *ovipneumoniae* (MO) at a final protein concentration of 14.3 and 10 μg/mL, respectively, for 24 h. Total RNA was prepared from these samples and gene expression was measured by Nanostring nCounter. Total RNA counts for each gene were normalised against the geometric mean counts of the three house-keeping genes (*GUSB*, *RPL15*, *HPRT1*). The gene expression for both pre- and post-vaccination (week 6) were obtained by dividing counts for antigen-stimulation by counts for PBS. Change in the expression of immune responsive genes are shown as the ratios of normalised RNA counts post-vaccination and normalised RNA counts pre-vaccination. The mean ratio of each group (*n* = 12) is presented here. Different letters written in superscript indicate significant differences (*P* < 0.05) between the groups, while the same letter indicates no difference.

A preliminary titration experiment was performed to determine the optimal concentration of RNA to use for the Plex nCounter runs. The expression of several genes in antigen-stimulated blood cells was modulated in animals vaccinated with antigens formulated with the different adjuvants compared to animals given antigens alone (Fig 7). Levels of mRNA for *CD40*, *IL22*, *IL2RA*, and *TGFB1* genes were higher in *M*. *ovipneumoniae*-stimulated whole blood cells from animals administered vaccines formulated with C18Brar or Quil-A compared to those given antigens alone (*P* < 0.05, Fig 7). Emulsigen-D and Alhydrogel + Quil-A adjuvants also stimulated higher levels of *TGFB1* (*P* < 0.05).

In contrast, *CD40* was downregulated in *M*. *haemolytica*-stimulated whole blood cells from animals vaccinated with antigens formulated with C18Brar compared to antigens alone (*P* < 0.05, Fig 7). Similarly, the expression of *IL2* and *IL2RA* and *BLIMP1* genes were downregulated in the Alhydrogel + Quil-A group (*P* < 0.05, Fig 7). The mean expression of *IFNG*, *IL13*, and *IL17A* genes was higher in animals vaccinated with antigens formulated with adjuvants compared to antigens alone, however, these differences were not statistically significant due to large animal-to-animal variation in responses.

## Discussion

*M*. *ovipneumoniae* is a primary infectious agent and pre-disposes sheep to secondary infections such as *M*. *haemolytica*. Strong humoral immune responses are often considered effective for providing protection against *M*. *haemolytica* and *M*. *ovipneumoniae* infections in sheep, and adjuvants that elicit strong antibody responses have been used in vaccines developed against pneumonia. However, recent reports suggest that cell-mediated immune responses could also play a crucial role in clearing these pathogens from the lungs of sheep and provide immunity against infection. Hence, adjuvants that promote both antibody and cellular immunity are needed for effective vaccines against ovine pneumonia. Ours is the first study contrasting the adjuvanticity of trehalose glycolipids to other veterinary adjuvants in *in vivo* vaccination studies with veterinary pathogens, and we have demonstrated that inactivated *M*. *haemolytica* and *M*. *ovipneumoniae* whole cell antigens formulated with C18Brar induced both humoral- and cell-mediated immune responses in sheep.

The *M*. *haemolytica* antibody titres elicited by animals vaccinated with C18Brar were significantly greater than those elicited by Quil-A or Alhydrogel + Quil-A at weeks 9 and 13. The antibody titres against *M*. *ovipneumoniae* were higher for C18Brar vaccinated animals at week 13 compared to those vaccinated with Emulsigen-D or Alhydrogel + Quil-A. Moreover, enhanced antigen-specific antibody responses to *M*. *ovipneumoniae* whole cell antigens were persisting long term and still evident at 31 weeks when C18Brar was used to augment immune responses. No IgA responses were observed in the current studies and only weak to moderate IgM responses were observed. While IgM and IgA antibodies play important roles in providing protective immunity against other invading pathogens at mucosal surfaces [63, 64], the role of these antibody isotypes in providing protective immunity against *M*. *haemolytica* and *M*. *ovipnuemoniae* appears to be limited [65–67]. Three of the four adjuvants studied, including C18Brar, stimulated the production of antibodies against LKT, which is a key virulence factor in respiratory disease in ruminants. Focussing on generating antibodies against LKT to achieve protection against *M*. *haemolytica* is a worthwhile strategy [12, 13], although the different serotypes of leukotoxin producing *M*. *haemolytica* makes it challenging to develop a vaccine against multi-variant leukotoxins [10, 11].

The ability of C18Brar to consistently stimulate strong antibody titres against both pathogens make it a promising adjuvant, particularly as the IgG antibody titres elicited by C18Brar were greater than or similar to those elicited by Quil-A and Emulsigen-D, two adjuvants that were previously shown to promote strong humoral response in veterinary vaccines [53, 68]. These findings are significant for the development of vaccines against ovine pneumonia since strong IgG antibody-responses to both *M*. *haemolytica* and *M*. *ovipneumoniae* antigens are thought to be crucial for neutralizing ovine pneumonia causing pathogens at mucosal surfaces and are likely to be the most important antibody isotype involved in providing protective immunity against pneumonia infection in sheep [32, 69, 70]. It should also be noted that whole cell antigen vaccines are often highly immunogenic and self-adjuvanting due to the presence of components such as lipopolysaccharide (LPS), DNA and RNA [71]. Thus, it was not surprising that in our study the control vaccine formulated with antigens alone stimulated antibody responses. However, the antibody responses were markedly higher in animals given vaccines formulated with some of the adjuvants, thus demonstrating the role, that additional adjuvants can have in augmenting vaccine-induced immune responses. The ability of C18Brar to elicit antibodies against LKT was also encouraging.

In addition to antibody titres, the ability of adjuvants to shape cellular immune responses is critical for the development of effective vaccines, particularly for those targeting intracellular pathogens [72, 73]. The results presented here demonstrate that C18Brar and the other adjuvants induced varying degrees of cell-mediated immune responses to *M*. *haemolytica* and *M*. *ovipneumoniae* whole cell antigens, as indicated by enhanced production of antigen-specific expression of IL-17A in whole blood *in vitro* assays. The response to C18Brar was broadly similar to that promoted by Quil-A, a known potent adjuvant, and is consistent with earlier studies demonstrating the ability of trehalose glycolipids to mediate Th-17 immune responses when using a variety of antigens [74]. All other adjuvants in our present study induced varying degrees of antigen-specific IL-17A responses to *M*. *haemolytica* and *M*. *ovipneumoniae*, with Emulsigen-D being the least effective.

IL-17A has been identified as a key player in providing protective immunity against intracellular pathogens, as well as at mucosal surfaces, and may be critical for vaccine-induced memory responses against infectious disease [75, 76]. Mycoplasma infections can result in IL-17 production in the lungs [31], leading to neutrophil recruitment, which is important for lung defence against the infection [28]. The ability of C18Brar to elicit Th-17-mediated immunity is therefore encouraging. Studies have demonstrated that IL-17 contributes to the clearance of bacterial pathogens such as *Pseudomonas aeruginosa*, *Klebsiella pneumoniae*, and *Streptococcus pneumoniae* in the respiratory tract [77, 78]. During *M*. *haemolytica* infection high levels of IL-17 in the lungs can lead to the uncontrolled infiltration of neutrophils and has been attributed to lung damage in mice, goats, and big horn sheep [26, 79, 80], although IL-17 does not seem to cause any damage in domestic sheep [80]. These studies suggest that IL-17 could help in clearing bacterial pathogens, particularly during early stages of respiratory infection, thus providing mucosal immunity during pneumonia. In the current study, IL-17A responses induced by a C18Brar formulated vaccine against *M*. *haemolytica* and *M*. *ovipneumoniae* could potentially enhance protective immunity against natural infection in sheep. Further studies will be needed to confirm this possibility.

Compared to antigen alone, none of the adjuvants appeared to stimulate significant increases in antigen-specific IFN-γ responses. It has been shown that blood cells stimulated *in vitro* for 48 h produce high levels of IFN-γ, possibly due to accumulation of the cytokine in the culture over time [81]. Measuring IFN-γ after a shorter incubation period than the 40 h used in our study may have revealed some difference in levels of IFN-γ stimulated by each adjuvant. Alternatively, it might be that none of the adjuvants increased levels of IFN-γ beyond that elicited in response to *M*. *haemolytica* and *M*. *ovipneumoniae* antigens alone.

The characterization of immune pathways in livestock species is often hampered due to lack of suitable immunological reagents. However, we found that the nCounter platform (Nanostring technology) allowed us to quantify expression of immune responsive genes. From these studies it was observed that several immune effector genes were modulated in the antigen-stimulated blood cells from animals vaccinated with antigens formulated with C18Brar compared with animals given antigens alone. The expression of *CD40*, a co-stimulatory protein expressed on APCs with an essential role in APC activation [82], was significantly increased in the animals vaccinated with the vaccine antigens formulated with C18Brar compared to antigens alone. Levels of mRNA for *IL22*, *IL2RA*, and *TGFB1* were all increased in the C18Brar adjuvant group after stimulation with *M*. *ovipneumoniae* antigen compared with antigen alone. *TGFB1* expression was also increased in the Emulsigen-D and the Alhydrogel + Quil-A groups. *IL22* and *IL2RA* promote T-cell-mediated immune responses [83–85], while *TGFB1* plays a crucial role in regulating T cell-mediated immune responses through a complex interplay of cytokines such as IL-6, IL-21, IL-22, and IL-23 to promote Th17 responses at mucosal surfaces [86–88]. The upregulation of *IL22* gene expression in response to *M*. *ovipneumoniae* antigens was noticeably strong in the C18Brar group. While the role of IL-22 in protection against respiratory pathogens such as *M*. *haemolytica* and *M*. *ovipneumoniae* is not clear [27, 30], IL-22 has been shown to modulate immune responses during infection at mucosal surfaces [78, 89, 90]. Overall, our findings suggest that the animals vaccinated with antigens formulated with C18Brar adjuvant produced increased cellular IL-17A responses as well as enhanced *M*. *ovipneumoniae* antibody responses, which were potentially augmented by the upregulation of immune response genes, in particular *CD40*, *IL22*, and *IL2RA*. Animals vaccinated with Quil-A also showed a similar profile of increased *CD40*, *IL22*, *IL2RA* and *TGFB1* transcription together with enhanced antibody and IL-17A responses to *M*. *ovipneumoniae* antigens.

Several genes in the adjuvant groups had slightly lower expression levels in response to *M*. *haemolytica* antigens compared to the antigen alone group. The expression of *CD40* in response to *M*. *haemolytica* antigens was downregulated in the C18Brar group, although this gene was upregulated in response to *M*. *ovipneumoniae*. The expression of *IL2*, *IL2RA*, and *BLIMP-1* were downregulated in the Alhydrogel + Quil-A vaccinated animals. *BLIMP-1* plays an important role in B-cell development, plasma cell differentiation and antibody production [91, 92]. It is worth noting that *M*. *haemolytica*-specific antibody titers in the animals vaccinated with Alhydrogel + Quil-A were not statistically different from the antigen alone group at any time point during the trial. A possible explanation for the observed downregulation of gene expression in response to *M*. *haemolytica* in the blood cultures might be due to the presence of LPS in the heat-killed *M*. *haemolytica* preparation. LPS may have elicited strong immune responses masking the effect of the adjuvants on immune cells.

The expression of several other immune genes including *IFNG*, *IL13*, and *IL17* genes were highly modulated in the adjuvant groups, but the observed differences were not statistically significant. A high level of variability in immune responses is often observed in outbred mammalian species [93], and for each adjuvant there were both high and low responding animals, which would have contributed to the high degree of variability within each adjuvant group. Nevertheless, the data presented here suggest that the same antigen modulates immune responses differently in sheep when formulated with different adjuvants, and that C18Brar augments the immune response towards *M*. *haemolytica* and *M*. *ovipneumoniae*. Moreover, the simple and low-cost chemical synthesis of C18Brar make it a particularly attractive adjuvant for use in veterinary vaccines where cost of goods is a key consideration. C18Brar is also readily prepared as a homogenous pure compound of defined structure [38]. This provides further advantages over adjuvants that are more difficult to obtain in pure form and which may be heterogeneous in nature.

A proportion of animals administered C18Brar, Emulsigen-D or Quil-A adjuvants developed site reactions and the animals from these adjuvant groups also showed enhanced humoral- and cell-mediated immune responses compared to animals given antigen alone. However, the observed lumps were transient in nature and disappeared during the trial and there were no animal welfare concerns. Other studies have also shown that a local site reaction to vaccination is a predictive sign of a desirable immune response [94–96]. Notwithstanding, further studies will be undertaken with different doses of C18Brar in the vaccine preparations to determine a dose of adjuvant that stimulates strong immune responses with minimal reactivity at the vaccination site. The use of the C18Brar adjuvant in vaccines tested in sheep naturally infected with pneumonia will establish if this class of adjuvant provides enhanced protective immunity against *M*. *ovipneumoniae* by inducing both a strong antibody and a balanced Th17 immune response.

### Summary

We evaluated the ability of the trehalose glycolipid adjuvant C18Brar to promote both humoral- and cell-mediated immune responses to *M*. *haemolytica* and *M*. *ovipneumoniae* whole cell antigens. C18Brar promoted strong antigen-specific antibody as well as Th17 immune responses to *M*. *haemolytica* and *M*. *ovipneumoniae*. Levels of serum IgG antibody and IL-17A release from antigen-simulated blood cultures were comparable to or higher than levels stimulated by a known potent adjuvant, Quil-A, with strong and long-lasting *M*. *ovipneumoniae* antibody titres being observed upon the augmentation of the vaccine with both of these adjuvants. Notably, C18Brar, but not Quil-A, led to significant increases in *M*. *haemolytica* antibody titres. Investigations into gene activation pathways suggested that the upregulation of genes including *CD40*, *IL22*, *IL2RA*, and to a lesser extent, *TGFB1*, might be partially responsible for the enhanced C18Brar- or Quil-A-adjuvanted immune responses to *M*. *ovipneumoniae*. Taken together, these results suggest that trehalose glycolipids have much potential as adjuvants for veterinary vaccines. Further investigations into the optimal dose of C18Brar and the potential of other members of the lipidated-brartemicin family of ligands to act as veterinary vaccine adjuvants are also warranted.

## Supporting information

S1 File Detailed procedure to synthesize C18Brar. (DOCX)

S1 Table List of genes analysed by Nanostring nCounter. (DOCX)

S1 Data Nanostring probe sets sequences. (XLSX)
