# Supplementary material for: The trehalose glycolipid C18Brar promotes antibody and T-cell immune responses to Mannheimia haemolytica and Mycoplasma ovipneumoniae whole cell antigens in sheep
Source: PLoS One. 2023 Jan 19;18(1):e0278853. doi: 10.1371/journal.pone.0278853 (PMC9851559; doi:10.1371/journal.pone.0278853)
Supplement: S1 File — (DOCX) [file pone.0278853.s001.docx]

**The trehalose glycolipid C18Brar promotes antibody and T-cell immune responses to *Mannheimia haemolytica* and *Mycoplasma ovipneumoniae* whole cell antigens in sheep**

Sandeep K. Gupta^1,*^, Natalie Parlane^1^, Benjamin Bridgeman^1^, Amy T. Lynch^2^, Emma Dangerfield^2^, Mattie Timmer^2^, Bridget Stocker^2,*^, D. Neil Wedlock^1^

**Supplementary Information**

**Detailed procedure to synthesize C18Brar**

Before use, methanol (Fisher), ethyl acetate (Pacific Sphere Limited) and petroleum ether (Vigor Sphere Pte Limited) were distilled under reduced pressure. Toluene (Scharlau) was dried and stored over Na wire. Dichloromethane (Fisher) was distilled from P_2_O_5_ (Reidel-de Haën) under an argon atmosphere. Dowex-H^+^ (Dowex® 50WX8, 100-200 mesh, Serva) was activated by washing with 1.0 M HCl, water (until filtrate was neutral), and methanol before use. Pyridine (Carlo Erba), NH_4_Cl (Carlo Erba), NaHCO_3_ (Pure Science), NaCl (Pure Science), MgSO_4_ (Pure Science), EDCI (Chem-Impex) and DMAP (Aldrich) were used as received. 2,2',3,3',4,4'-*hexa*-*O*-trimethlsilyl-α,α'-ᴅ-trehalose **S1** and 4-octadecyloxy-benzoic acid **S2** were prepared according to literature procedures (Johnson, 1992; Foster et al., 2018). Solvents were removed by evaporation under reduced pressure. Reactions were monitored by TLC with Macherey-Nagel silica gel-coated plastic sheets (0.20 mm with fluorescent indicator UV_254_) via detection by UV absorption (254 nm) and dipping in a solution of 10% H_2_SO_4_ in EtOH followed by charring. Column chromatography was performed using silica gel (40-63 µm, Roth). Nuclear magnetic resonance (NMR) spectra were recorded at 20°C in CDCl_3_ (Aldrich) or C_5_D_5_N (Apollo Scientific) using a Varian INOVA operating at 500 or 600 MHz. Chemical shifts are given in ppm (δ) relative to residual solvent peaks. NMR peak assignments were made using correlated spectroscopy (COSY), HSQC and HMBC 2D experiments.

**2,2',3,3',4,4'-*Hexa*-*O*-trimethylsilyl-6,6'-di-(4-octadecyloxy-benzoyl)-α,α'-ᴅ-trehalose (S3).** 2,2',3,3',4,4'-*hexa*-*O*-trimethlsilyl-α,α'-ᴅ-trehalose **S1** (2.30 g, 3.0 mmol) was co-evaporated with anhydrous toluene (2 x 30 mL) and then dissolved in anhydrous toluene (45 mL) and placed under an atmosphere of argon. EDCI (4.61 g, 24.1 mmol), DMAP (789 mg, 6.5 mmol) and carboxylic acid **S2** (4.64 g, 11.9 mmol) were then added. The mixture was stirred at 70°C for 5 days, with additional EDCI (1.71 – 2.33 g, 9.0 – 12.2 mmol) and carboxylic acid **S2** (1.93 - 2.32 g, 4.7 – 5.9 mmol) being added after 24 hours and on day 3. Upon reaction completion, the mixture was cooled and diluted with hot ethyl acetate (1.75 L). The organic layer was washed with saturated NH_4_Cl (800 mL), saturated NaHCO_3_ (800 mL) and brine (800 mL), dried (MgSO_4_), filtered and, concentrated. The resultant yellow oil was purified by gradient flash silica gel chromatography (petroleum ether → 4:1 petroleum ether:ethyl acetate, *v/v*) to give the title compound as a white solid (2.64 g, 1.7 mmol, 59%). R*_f_* = 0.61 (Petroleum ether:EtOAc, 19:3, *v/v*); ^1^H-NMR (500 MHz, CDCl_3_) δ 8.33 (d, *J*_2',3'_ = 8.8 Hz, 4H, H-2'), 7.25 (d, *J*_2',3'_ = 8.8 Hz, 4H, H-3'), 5.32 (d, *J*_1,2_ = 3.1 Hz, 2H, H-1), 4.88 (dd, *J*_6a,6b_ = 12.0 Hz, *J*_5_*,*_6a_ = 2.4 Hz, 2H, H-6a), 4.59 (dd, *J*­_6a, 6b_ = 12.2 Hz, *J*_5, 6b_ = 3.4 Hz, 2H, H-6b), 4.46 (dt, *J*_4,5_ = 9.4 Hz, *J*_5,6a_ = *J*_5,6b_ = 2.8 Hz), 4.35 – 4.29 (m, 6H, H-3 & H-5'), 3.99 (t, *J*_2,3_ = *J*_3,4_ = 9.0 Hz, 2H, H-4), 3.84 (dd, *J*_2,3_ = 9.3 Hz, *J*_1,2_ = 3.0 Hz, 2H, H-2), 2.13 (p, *J*_5',6'_ = *J*_6',7'_ = 7.1 Hz, 4H, H-6'), 1.82 – 1.76 (m, 4H, H-7'), 1.65 -1.59, m, 56H, H-8' – H-21'), 1.22 (t, *J*_21',22'_ = 6.7 Hz, 6H, H-22'), 0.52, 0.49, 0.46 (3 s, 54H, CH_3_ TMS); ^13^C-NMR (125 MHz, CDCl_3_) δ 166.4 (C=O), 163.2 (C-4'), 131.9 (C-2'), 122.3 (C-1') 114.2 (C-3'), 94.8 (C-1), 73.8 (C-3), 72.9 (C-2), 72.1 (C-4), 71.0 (C-5), 68.4 (C-5'), 63.6 (C-6), 29.3 (C-6'), 26.2 (C-7'), 32.1, 29.85, 29.83, 29.81, 29.75, 29.72, 29.53, 29.52, 22.8 (C-8' – C-21') 14.3 (C-22'), 1.3, 1.1, 0.4 (TMS).

**6,6'-Di-(4-octadecyloxy-benzoyl)-α,α'-ᴅ-trehalose (C18Brar).** To protected di-ester **S3** (973 mg, 640 µmol) dissolved in CH_2_Cl_2_:Methanol (3:2, *v/v*, 25 mL) was added Dowex-H^+^ (121 mg). The mixture was stirred slowly at room temperature for 3 h, at which time it was diluted with pyridine (20 mL) and filtered, washing the resin with hot pyridine (3 x 10 mL). The filtrate was concentrated to give a white solid. This crude product was dissolved in boiling ethanol (200 mL) and then cooled. The resultant white precipitate was collected by filtration, washing with small portions of ice-cold ethanol, and dried in a desiccator, to give the title compound as a white solid (659 mg, 606 µmol, 95%). Characterisation data was consistent with previous report (Foster et al., 2018).

**Vaccine formulation**

To prepare the adjuvant emulsion, C18Brar [3.75 mg/vaccine dose (2.5 mL)] was crushed to a fine powder, then dissolved in mineral oil (225 μL/dose) by mixing (vortexing). Tween-80 (25 μL/dose), followed by PBS (1000 μL/dose) were added and the resulting emulsion mixed with vortexing and sonication for 30 mins and stored at 4 °C prior to use. For vaccine formulation, bacterial antigens in PBS were diluted 1:1 with the adjuvant preparation to form a homogeneous emulsion 1 day prior to vaccination. Average droplet sizes for emulsions were measured using dynamic light scattering (DLS) with a ZetasizerNano ZS (Malvern Instruments Ltd, Malvern, Worcestershire, UK) at 20 °C. Data was analysed using Malvern Dispersion Technology Software. Analysis of the adjuvant emulsions by DLS revealed a bimodal distribution of droplet sizes with average diameters of 100±22 nm and 370±92 nm, although larger aggregates were also observed.


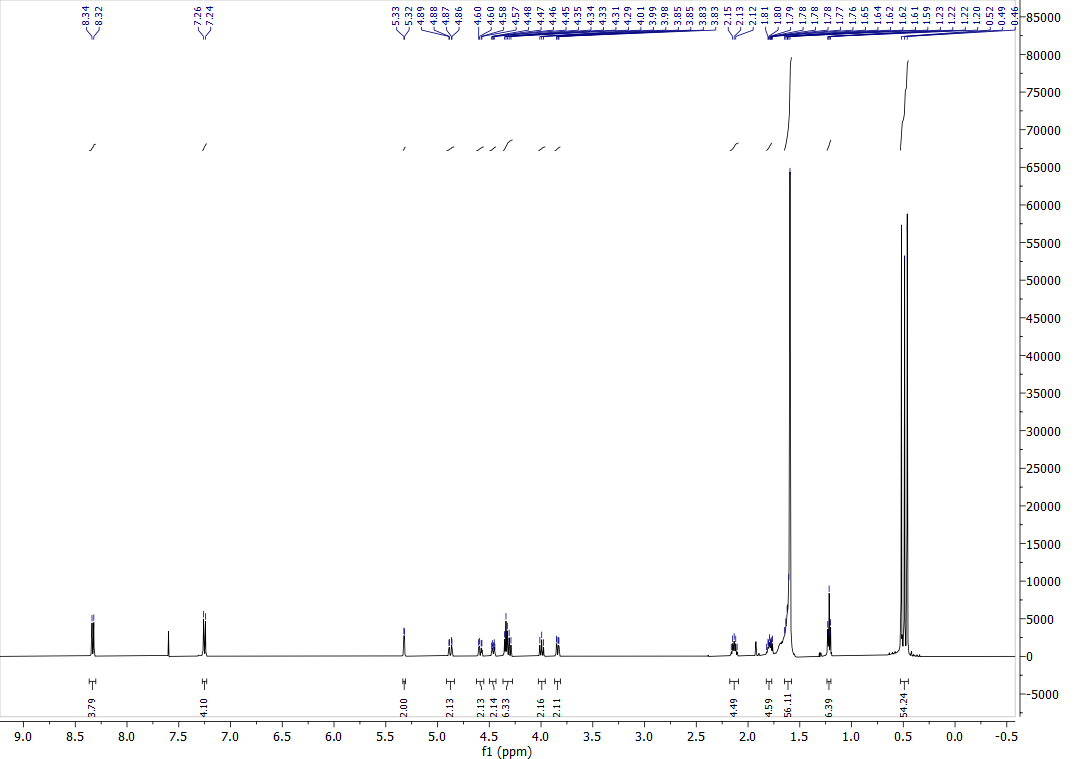


**Figure S1. 2,2',3,3',4,4'-*Hexa*-*O*-trimethylsilyl-6,6'-di-(4-octadecyloxy-benzoyl)-α,α'-ᴅ-trehalose (S3)** ^1^H-NMR (500 MHz, CDCl_3_)

**Figure S2. 2,2',3,3',4,4'-*Hexa*-*O*-trimethylsilyl-6,6'-di-(4-octadecyloxy-benzoyl)-α,α'-ᴅ-trehalose (S3)**^13^C-NMR (125 MHz, CDCl_3_)


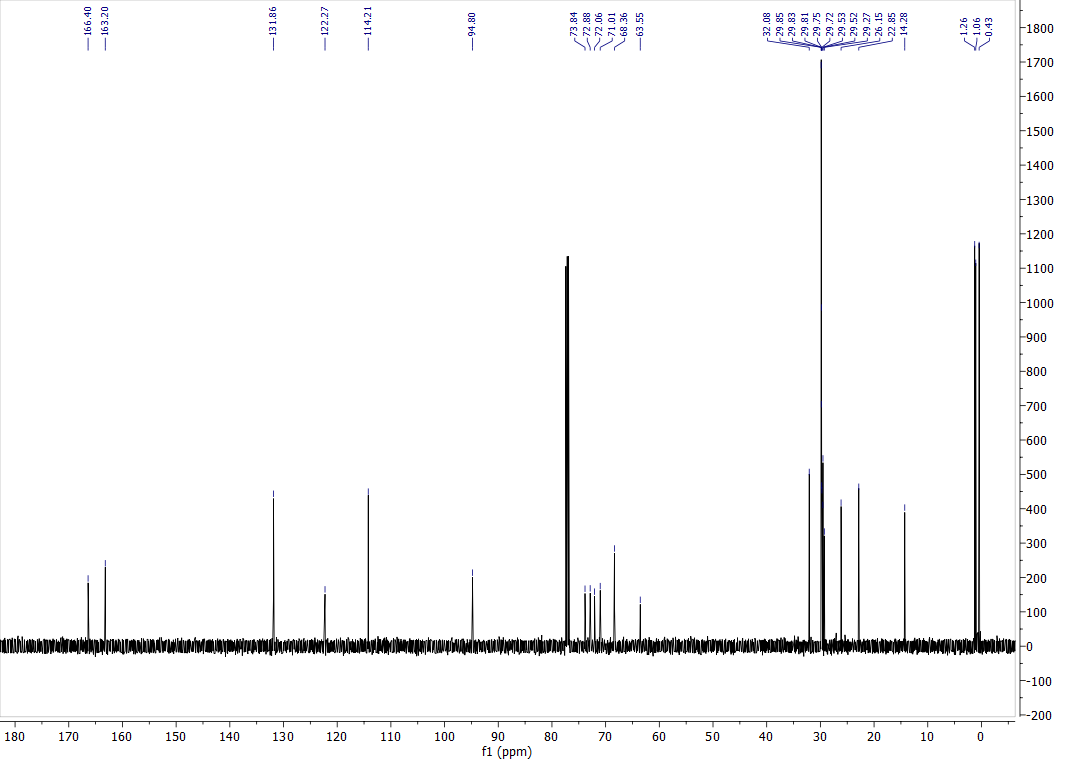

**Figure S3. 6,6'-Di-(4-octadecyloxy-benzoyl)-α,α'-ᴅ-trehalose (C18Brar)**^1^H-NMR (500 MHz, pyridine-d_5_)

**Figure S4. 6,6'-Di-(4-octadecyloxy-benzoyl)-α,α'-ᴅ-trehalose (C18Brar)**^13^C-NMR (125 MHz, pyridine-d_5_)

**Figure S5.** Antibody responses to leukotoxin in the vaccinated animals. Serum IgM antibody responses to *M. haemolytica* and *M. ovipneumoniae* at 4 weeks after initial vaccination in animals vaccinated with either Antigens alone (Ags), antigens formulated with Quil-A (QA), Emulsigen-D (ED), Aldhydrogel + QuilA (AGA), or C18Brar. Antibody responses were measured in serum samples using ELISA. No significant differences were observed between the groups.

**References**

Johnson, D. A., 1992. Simple procedure for the preparation of trimethylsilyl ethers of carbohydrates and alcohols. *Carbohydr. Res.* *237*, 313-318.

Foster, A.J., Nagata, M., Lu, X., Lynch, A.T., Omahdi, Z., Ishikawa, E., Yamasaki, S., Timmer, M.S.M., Stocker, B.L., 2018. Lipidated Brartemicin Analogues Are Potent Th1-Stimulating Vaccine Adjuvants. *Journal of Medicinal Chemistry 61*, 1045-1060.
