# Supplementary material for: The trehalose glycolipid C18Brar promotes antibody and T-cell immune responses to Mannheimia haemolytica and Mycoplasma ovipneumoniae whole cell antigens in sheep
Source: PLoS One. 2023 Jan 19;18(1):e0278853. doi: 10.1371/journal.pone.0278853 (PMC9851559; doi:10.1371/journal.pone.0278853)
Supplement: S1 Table — (DOCX) [file pone.0278853.s002.docx]

**Table S1.** List of genes analysed by Nanostring nCounter.

| **Gene name** | **Pathway/cell function** |
| --- | --- |
| GUSB | House-keeping genes |
| RPL15 |  |
| HPRT1 |  |
| Ovine Lymphocyte antigen class I (OLA-I) | T- and B-cells activation markers |
| Ovine Lymphocyte antigen class II (OLA-II) |  |
| CD86 |  |
| CD28 |  |
| CD40 |  |
| CD40LG |  |
| CTLA4 |  |
| B lymphocyte-induced maturation protein-1 (BLIMP-1) |  |
| IL2RA |  |
| ITGAM | Cytokine responses |
| TNF |  |
| CCL2 |  |
| CXCL10 |  |
| IL2 |  |
| IL13 |  |
| IFNG |  |
| IL17A |  |
| IL22 |  |
| IL4 |  |
| TGFB1 |  |
| IL10 |  |
